# One-shot technology for three-dimensional imaging of large animals: perspectives for ruminant management

**DOI:** 10.1093/tas/txae018

**Published:** 2024-02-09

**Authors:** Yannick Le Cozler, Maxime Dumesny, Jean-Michel Delouard, Laurent Delattre, Thibault Luginbühl, Philippe Faverdin

**Affiliations:** PEGASE, INRAE, Institut Agro Rennes-Angers, 35590 Saint Gilles, France; PEGASE, INRAE, Institut Agro Rennes-Angers, 35590 Saint Gilles, France; 3D Ouest, 5 Rue de Broglie, 22300 Lannion, France; 3D Ouest, 5 Rue de Broglie, 22300 Lannion, France; 3D Ouest, 5 Rue de Broglie, 22300 Lannion, France; PEGASE, INRAE, Institut Agro Rennes-Angers, 35590 Saint Gilles, France

**Keywords:** 3D images, cattle, depth camera, one shot

## Abstract

In numerous systems of animal production, there is increasing interest in the use of three-dimensional (3D)-imaging technology on farms for its ability to easily and safely measure traits of interest in living animals. With this information, it is possible to evaluate multiple morphological indicators of interest, either directly or indirectly, and follow them through time. Several tools for this purpose were developed, but one of their main weaknesses was their sensitivity to light and animal movement, which limited their potential for large-scale application on farms. To address this, a new device, called Deffilait3D and based on depth camera technology, was developed. In tests on 31 Holstein dairy cows and 13 Holstein heifers, the values generated for most measured indicators were highly repeatable and reproducible, with coefficients of variation lower than 4%. A comparison of measurements obtained from both Deffilait3D and the previous validated system, called Morpho3D, revealed a high degree of similarity for most selected traits, e.g., less than 0.2% variation for animal volume and 1.2% for chest depth, with the highest degree of difference (8%) noted for animal surface area. Previously published equations used to estimate body weight with the Morpho3D device were equally valid using Deffilait3D. This new device was able to record 3D images regardless of the movement of animals and it is affected only by direct daylight. The ongoing step is now to develop methods for automated analysis and extraction from images, which should enable the rapid development of new tools and potentially lead to the large-scale adoption of this type of device on commercial farms.

## Teaser

Thanks to three-dimensional imaging based on one-shot technology; it is possible to determine morphological traits, body weight, surface, or volume of dairy cows. The upcoming automation of measurements opens up prospects for high-throughput applications and analyzes on farms.

## Introduction

Early attempts to monitor changes in livestock animals using imaging technologies were initially based on two-dimensional (2D) imaging (Schofield et al., 1999). Two-dimensional camera-based approaches gave significant results in estimating body area and/or other morphological traits (Tasdemir et al., 2011; Ozykaya et al., 2016; Yan et al., 2019; Weber et al., 2020a, b). However, the process of converting a three-dimensional (3D) object into a 2D image encountered significant challenges associated with image distortion and deformation. Research focus thus shifted to 3D imaging technologies (Gauthier, 2005), which are, such as 2D imaging technology, non-intrusive and capture partially or totally the external envelope of a living animal, depending on the number of cameras used (Wang et al., 2018; Le Cozler et al., 2019a; Martins et al., 2020; Li et al., 2022). With this approach, it is possible to describe the morphological characteristics of an animal, or changes in such characteristics over time, without any risk to the handler or the animal, even if the animal is vigorous and/or dangerous. When used with appropriate software, 3D imaging has great potential for quantifying or estimating technical indicators of interest such as body length or body condition score, live weight, or lameness detection, among others (Bewley et al., 2008; Halachmi et al., 2008; Fischer et al., 2015; Pezzuolo et al., 2018; Le Cozler et al., 2020b, 2022a). Past work by our group has demonstrated the added value of 3D imaging for monitoring morphological traits to which it was rarely possible to have direct access, such as surface area and volume. From these indicators, it then becomes possible to estimate body weight (BW) in both adults and young animals (Le Cozler et al., 2019a, 2022b; Wang et al., 2023). We have also shown that information from 3D-imaging technology can be used to study the nature of weight gain during lactation (Xavier et al., 2022a), but also to estimate the chemical composition of animals (Xavier et al., 2022b). For these earlier studies, we used an imaging device called “Morpho3D,” which is very accurate but requires specific conditions of use that are not always easy to obtain, including the absence of direct light and a complete lack of movement by the animals while recording images (5-s acquisition time).

To address these problems, and to improve the speed and ease of acquiring a large amount of phenotypic information, Li et al. (2022) developed a real-time point-cloud collection gantry system, which enables body measurements for active cattle on a ranch. Our group, instead, developed an approach based on the use of multiple depth cameras, triggered simultaneously (“one shot”), which can capture the external envelope of the animal via the coupling of the different image captures. With this technique, we hoped to develop a method that was less affected by animal movements during the acquisition time and was less dependent on direct light. To this end, we have created a new imaging device, called “Deffilait3D” that combines multiple simultaneously acquired images to create a 3D reconstruction of an animal. Here, we present the results of validation tests in which we compared values measured on Holstein cows using the Deffilait3D and Morpho3D systems, the measurements from the latter, previously validated, device used as reference (Le Cozler et al., 2019a). In addition to evaluating repeatability and reproducibility, we also assessed the accuracy of earlier equations developed with the Morpho3D device in estimating the BW of animals (Le Cozler et al., 2019b) using values collected from Deffilait3D.

## Materials and Methods

The experiment was conducted within the ANR Deffilait project, with procedures approved by the local ethics committee and the French Ministry of Higher Education, Research, and Innovation (reference number APAFIS 3122-2015112718172611).

### Experimental Design

The study was performed from March to June 2021 at the Méjusseaume experimental station of INRAE, Dairy Nutrition and Physiology unit (IE PL, 35560 Le Rheu, France (https://doi.org/10.15454/yk9q-pf68) under agreement for animal housing no. C-35-275-23). This facility has a herd of approximately 180 Holstein cows and their offspring (e.g., replacement heifers), producing an average of 8,500 kg of milk per cow per year. All animals were reared and managed under routine farm procedures consistent with French animal welfare regulations.

### Data Collection

**Figure 1.**
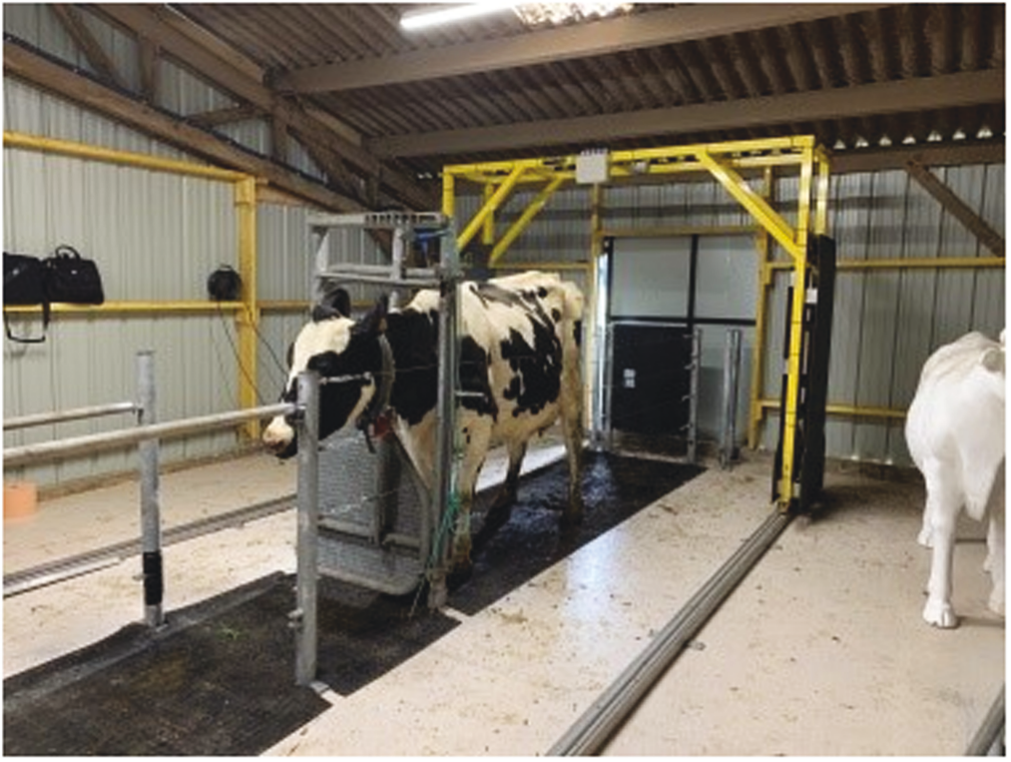
The Morpho3D device at the experimental milk production facility (IEPL).

**Figure 2.**
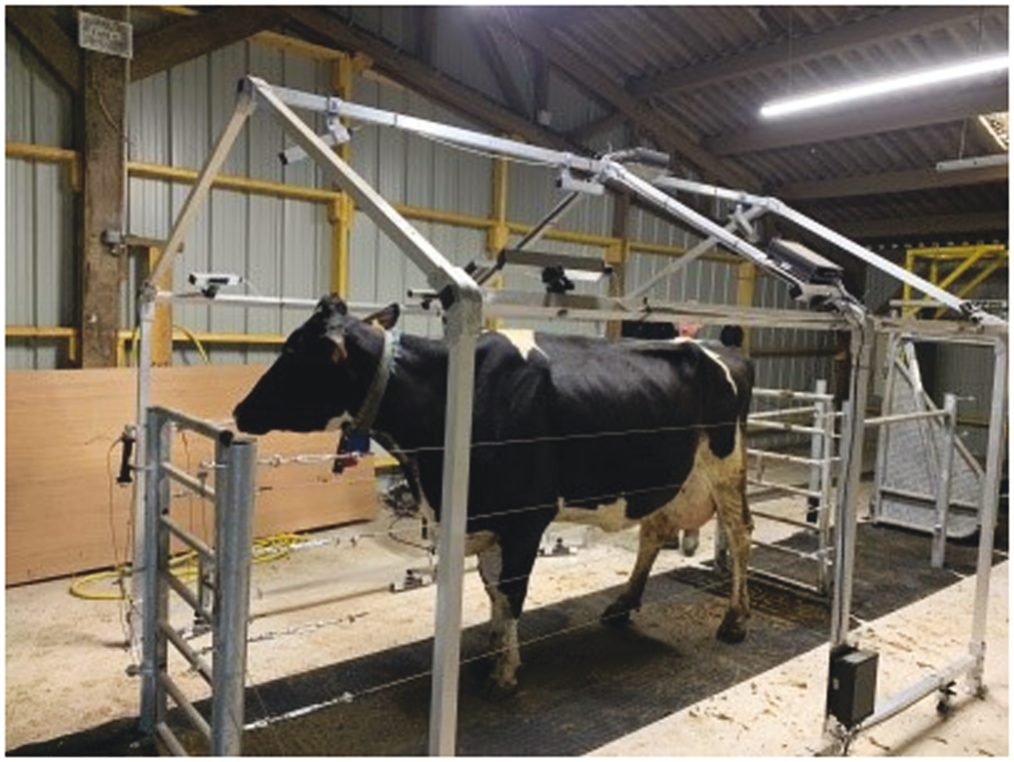
The Deffilait3D device at the experimental milk production facility (IEPL).

**Figure 3.**
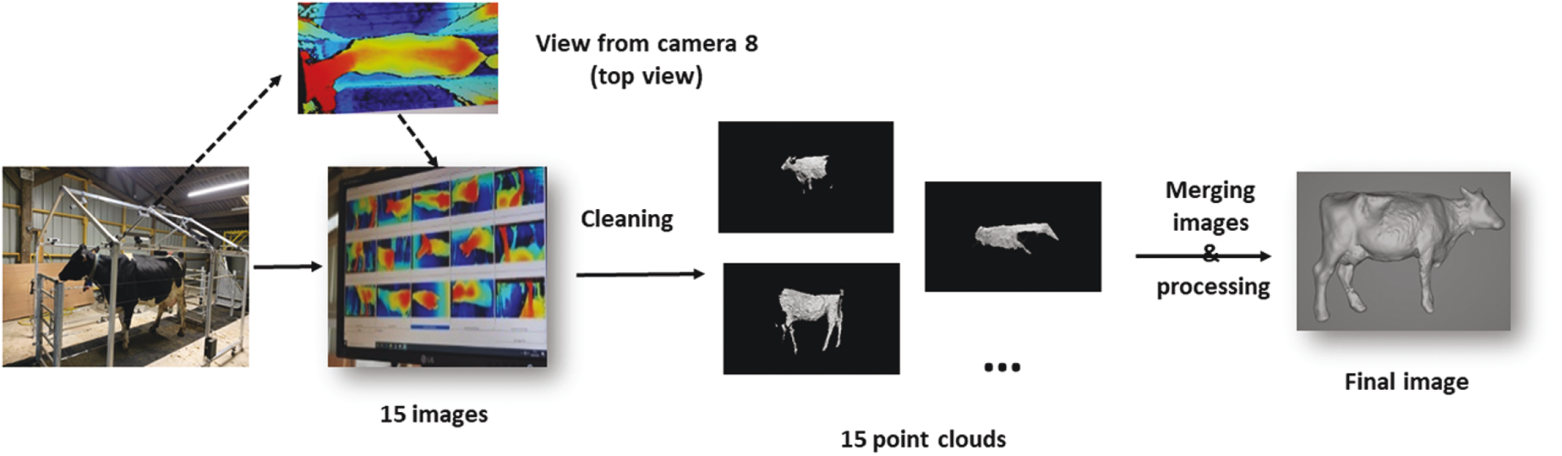
The different steps of image acquisition using the Deffilait3D device.

Two devices for the acquisition and processing of 3D images of whole animals are present in this experimental installation, both developed, and fine-tuned by the company 3DOuest (Lannion, France):

(1) Measurements obtained from the Morpho3D device were used as reference values (“gold standard”), since this system has been repeatedly tested, validated, and used since 2017 (Le Cozler et al., 2019a for more details; Figure 1). For this reason, no manual measurements, as traditionally performed and considered as “gold standards” for morphological traits (Heinrichs et al., 1992), were performed. Morpho3D device is a sliding acquisition system with five cameras distributed over the sides and top of the gate, each coupled to a laser projector. As the gate moves back and forth, each camera takes 80 images per second, giving a total of 2,000 images. The images of the laser strips projected onto the animals are captured by the corresponding camera and sent to a computer. During the image acquisition period (5 to 6 s), the animal must stand completely still. For this reason, four stainless steel cables are also used within the Morpho3D Scanner, on both sides of the cow, to secure the scanner and restrict cow movement. Cows can also be restrained by a self-locking headgate if necessary but as it becomes accustomed to the device and its movement, restraints are no longer necessary. After processing, a 3D reconstruction is generated, with a unique point-cloud representation of the whole animal. The surface normals are estimated from the point cloud and a screened Poisson surface reconstruction algorithm is applied to construct a triangulated mesh (Kazhdan and Hoppe, 2013), using the open-source software Meshlab (Cignoni et al., 2008).
(2) The Deffilait3D device was installed at the end of 2020. This prototype consists of a fixed metal frame, with 15 RGB-D sensors in total (Intel RealSense D415, Figure 2). The Intel RealSense D415 has a compact design, low cost, and low power usage (USB-powered). It is usually used in indoor environments, as sunlight may interfere with the infrared (IR) intensity of projector, leading to holes in the depth map. The RealSence D415 is depth camera and consists of a pair of depth sensors, an RGB sensor, and an infrared spotlight. This technology is a stereo system: it triangulates the depth (distance) for each pixel between the two sensors. The infrared projector helps the algorithm to find correspondence between the two images. For the animal reconstruction, in a passive stereo system, two RealSence D415 cameras, whose relative position and orientation are known, are used to capture the same scene. The two images are searched for corresponding pixels, and for each correspondence found, the depth can be estimated using camera parameters and triangulation. Here, the active stereo is used and aims to simplify the search for corresponding pixels by adding data with a light source emitting a pattern. Depth information is represented using false colors images, blue, and red indicate the distance from far (blue) to near (red). Each sensor is connected to a computer, which is itself connected to a terminal. The images taken simultaneously by these 15 devices are then combined to reconstruct a 3D image of the entire animal. A common coordinate frame is chosen (the upper camera frame) and coordinate frame transforms for every acquisition are established through a calibration procedure: a checkerboard pattern is shown to consecutive camera’ pairs, the relative positions of the cameras are estimated using this pattern and OpenCV calibration module. Once the depth maps are computed, a smoothing algorithm is applied (Gastal and Oliveira, 2011), this is mostly to compensate for the staircase effect caused by discretized depth values. The different steps of the procedure are presented in Figure 3. Unlike the Morpho3D device, this method does not require immobilization of the animal. However, four stainless steel cables are also used within the Deffilait3D Scanner, on both sides of the cow, to secure the scanner and restrict cow movement, but cows cannot be restrained by a self-locking head gate. If necessary, a bucket of pellets is sometimes used, especially during the first few passes, to reassure the animal (but also to reward it and encourage it to return). The speed of this device allows high-speed acquisition of 3D images, but can also be used to record almost continuously as the delay between photographic triggers can be reduced to a fraction of a second.

### Data

In the IEPL facility, the Morpho3D and Deffilait3D devices are located in the same room. 3D images of a given animal were then collected under the same conditions, a few seconds or minutes apart. In both cases, the images were analyzed using Metrux8α® software (3D Ouest, Lannion, France). When using Morpho3D device, the 3D acquisitions were not always fully exploitable due to the animal’s movements or the necessity to block the animal thanks to the self-locking head gate for example. As a result, images were sometimes cut off at the shoulder level and we developed then predicting model of total surface area (TS) or volume (TV) from partial surface area (PS) or volume (PV; Le Cozler et al., 2019b). When the image of the full animal is available, TS and TV are calculated using the automatic algorithm. When cutting off is necessary, PS and PV are measured by placing one plane at the tip of the shoulder blades and another at the rump (Le Cozler et al., 2019b).

Metrux8α® software has functions to quickly measure interactively:

(1) the partial volume (TV, respectively) of an animal;
(2) its partial or total surface area (PS or TS, respectively);
(3) height at the withers (WH), hip width (HW), buttocks width (WB), diagonal length (obtained by averaging the left and right diagonal lengths, i.e., between the tips of the buttocks and the tips of the shoulders), chest depth (CD), and heart girth (HG, see Le Cozler et al., 2019a for more details on locations). To these initial measurements that have already been presented in previous publications, we also added the abdomen circumference (AC).

These measurements are not automatic and the points of interest must be “manually clicked”. Thereafter, these measures are performed thanks to geometric computations (distances, curve lengths, etc.) based on feature points selected by the user. For the determination of the volume, the method presented by Mirtich (1996) is used.

The images acquired using the Deffilait3D and Morpho3D devices were of variable quality. Each image was therefore manually assigned a quality score varying from 0 to 4, with the following characteristics:

(1) 0: unusable point clouds, i.e., when there was too much movement by the animal;
(2) 1: reconstructed image usable for some measurements but still of poor quality (e.g., totally deformed legs);
(3) 2, 3, and 4 correspond respectively to average, good, and very good images, on which most or all measurements were feasible.

**Figure 4.**
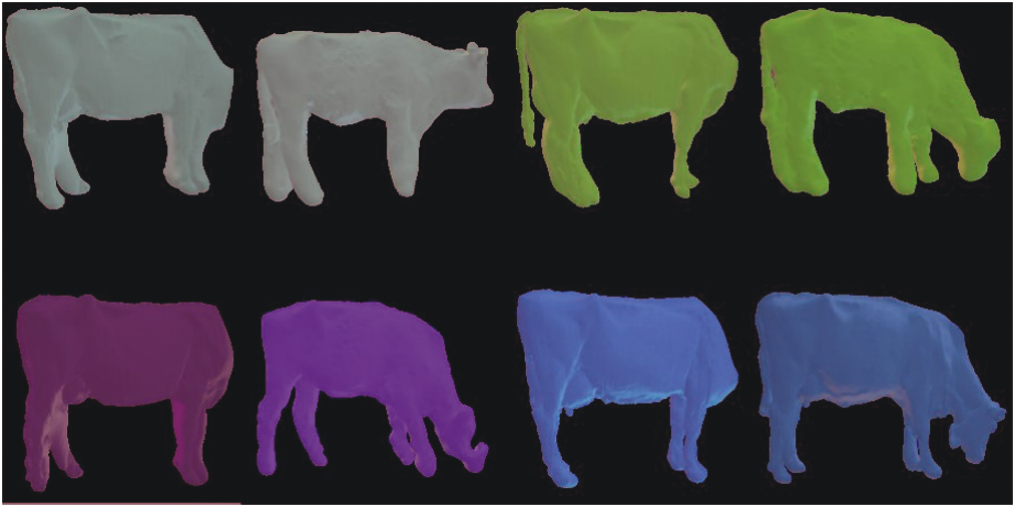
Ranking of 3D image quality, from 1 to 4 + (used for images obtained from Morpho3D and Deffilait3D devices).

We also added a “+” sign after each score to indicate whether the image of the animal was complete or not, e.g., with or without the head. Systematically, because of the differences between the gantries, images from the Deffilait3D device always had a “+” while those from the Morpho3D device did not. Examples of scoring are shown in Figure 4.

### Validation of the Deffilait3D Device

In order to test the validity and utility of the Deffilait3D device, we carried out a series of trials in which the repeatability and reproducibility of the device were assessed. Analysis was performed on 15 cows, in two trials

(1) Repeatability is “the closeness of agreement between successive results obtained with the same method on the same material under test and under the same conditions” (ISO Standard 3534). In this case, “under the same conditions” meant that the measurements were made with the same operator, the same equipment, in the same place, and in a very short time interval. In the context of points, an analysis of repeatability makes it possible to know if, for a given acquisition, the identification of the points for predicting, e.g., the same partial volume, is correct. For this purpose, eight cows were chosen, these cows being considered as representative of the variability in BW and size observed in this herd. The morphology and the chosen indicators (HW, WH, WB, diagonal length, PS, PV, CD, HG, and AC) were determined from a single image acquisition, from which the measurements were repeated five times by the same operator. In this way, we tested the repeatability of the point identification.
(2) Reproducibility is “the closeness of agreement between individual results obtained with the same method on the same test material under different conditions” (ISO Standard 3,534). Different conditions may be related to different operators or different locations, for example, and intermediate cases are possible, such as different operators working in the same location or measurements made with the same equipment but with a long interval between acquisitions. It is thus possible to define and calculate reproducibility between operators, between devices, or across days. Here, we tested intra-facility reproducibility, which evaluates how well the method is able to predict the same data in different acquisitions in a changing environment (i.e., with the animals in slightly different positions). The assessment takes into account the image capture by the scanner, the fusion of the 15 images, from the 15 pairs of sensors and laser used, image cleaning, reconstruction, and measurement. For the reproducibility test, eight cows were used, of which seven were different from those used in the repeatability tests. As in previous tests, they were considered to be representative of the variability observed in BW and size in this experimental herd. Each was subjected to five different sessions of image acquisition, taken at relatively short time intervals (10 to 20 min). For each image, the same measurements were captured as for the repeatability test, one time by the same operator.

### Comparison of the Deffilait3D and Morpho3D Devices

Once the repeatability and reproducibility of the Deffilait3D device were validated, its measurements were compared with those obtained from the Morpho3D device, which were considered the reference values (“gold standard”). Fifteen adult dairy cows were randomly selected and passed (“scanned”) through each device. These cows differed from those used in previous repeatability and reproducibility tests. Following the acquisition and reconstruction of the 3D images from each device, selected morphological measurements were determined and compared. For the sake of time, we limited our assessment to this panel of 15 cows and values of HW, WH, CD, HG, TV, and TS. In order to limit animal stress as much as possible and to avoid retaking measurements unnecessarily, the animals were physically restrained in a feed fence in the Morpho3D device. For the surface and volume measurements, TS and TV were then calculated from PS and PV, which correspond to the surface area and volume without the head and neck, using previously published prediction equations (Le Cozler et al., 2019a):


$$\begin{array}{lll} TV & (total\;volume)=\; & 1.0704\times PV(volume\;cutof\;f\;at\;the\;shoulder)+0.015 \end{array}$$

$$\begin{array}{lll} TS & (total\;surface\;area)=\; & 1.07\times PS(surface\;area\;cutof\;f\;at\;shoulder\;tip)+0.94 \end{array}$$

### Validation of Published Equations

In order to determine whether the Deffilait3D device could provide reliable and useful values from prediction equations established with the Morpho3D device, we tested the equation predicting BW on a group of 13 heifers, which represented a high degree of variation in BW measured on a weighing scale (440 to 550 kg). The morphological traits described above were determined for these animals, and the equation presented by Le Cozler et al. (2019a)—BW = 644 × TV + 408 × HW + 271 × WB—199—was then used to predict BW. Other equations for predicting BW are available but are not presented here (this one was selected because it gave the most accurate results).

### Statistical Analysis

Data were first visualized using the *ggplot2* package (v. 3.2.1, Wickham et al., 2019) of R software (R Core Team, 2018). Other statistical analyses were realized under R version 3.5.2 with “car” package for analysis of variance and regression, to compare values from Morpho3D and Deffilat3D devices (Fox and Weisberg, 2011). In general, measurement values may vary among the different 3D images used (five images in total for each cow) based on slight differences among images as well as differences in point identification within a single image. By definition, then, the reproducibility error encompasses the repeatability error of point identification and will therefore always be the higher value of the two. The parameters used to quantify repeatability and reproducibility were the standard deviations of point repeatability, σrp, and intra-facility reproducibility, σRi, which were calculated from the residuals of the one-factor analysis of variance (ANOVA) model (the cow effect). The ANOVA, as well as other repeatability and reproducibility calculations, were performed with R software (R Core Team, 2018). With this approach, existing morphological variations between cows are removed in the analysis. The coefficients of variation of point repeatability, CVrp, and intra-facility reproducibility, CVRi, are then expressed as follows:


$$\begin{array}{l} CVrp=\sigma rxr\times 100 \end{array}$$

$$\begin{array}{l} CVRi=\sigma RxR\times 100 \end{array}$$

where *xr* and *xR* are the respective means of the study variable in the populations used for the repeatability and reproducibility assessment.

The lower the coefficient of variation, the more repeatable, or reproducible the method or part of the method is.

From a mathematical point of view, repeatability (r) and reproducibility (R) are each defined by the value below which the absolute value of the difference between two individual results (x1 and x2) obtained under either repeatability or reproducibility conditions lies with a specified probability. If not otherwise specified, the probability is 95%.

i.e.,: P (|x1−x2|≤r)=0.95 and P (x1−x2≤R)=0.95

In other words, *r* and *R* represent in each case the arithmetic difference between two determinations with a 95% probability of not being exceeded. Repeatability and reproducibility are considered good if the CVs are below 4%, which was used as the reference value for this study (Fischer et al., 2015; Le Cozler et al., 2019a).

## Results

### Repeatability and Reproducibility Tests to Validate the “Deffilait3D” Device

The repeatability and reproducibility tests used a total of 40 (8 × 5) and 200 (8 × 5 × 5) images, respectively, with all being of sufficient quality (score of 3 + minimum). For all measurements, the coefficients of variation of point-related repeatability (CVrp) in the images were low (below 1.8%), with three CVrp values below 0.5% (HW, WB, AC; AC; Table 1). Coefficients of variation of reproducibility varied from 1.12% to 3.26%, with an average CVRi of 2.07%. The least reproducible indicators were partial surface area (3.26%) and complete volume (2.71%), and these were probably due to imperfect images, including poorly reconstructed limbs or heads. Concretely, for a cow with a volume of 764 L, the repeatability and reproducibility errors were 8.71 and 21.41 L, respectively. For the same cow with a total surface area of 7.20 m², the errors in repeatability and reproducibility of surface area were 0.13 and 0.23 m², respectively. Finally, for an average WH of 1.45 m, the repeatability error was 0.019 m and the reproducibility error was 0.026 m. The full repeatability and reproducibility tables for each cow used in the present experiment are available in Supplementary File S1.

**Table 1.** Errors and coefficients of variation of repeatability and reproducibility for morphological measurements using the Deffilait3D device

	Coefficient of variation, %		Error	
	Repeatability	Reproducibility	Repeatability	Reproducibility
Complete volume, L	1.18	2.71	8.71	21.14
Partial surface, m²	1.77	3.26	0.13	0.23
Hip width, m	0.48	1.12	0.0028	0.0064
Withers height, m	1.29	1.78	0.0186	0.0264
Chest depth, m	0.81	1.42	0.0064	0.0116
Heart girth, m	0.46	2.16	0.0118	0.047
Buttocks width, m	1.34	1.83	0.007	0.01
Diagonal length, m	0.76	2.55	0.013	0.0447
Abdominal circumference, m	0.46	1.8	0.0118	0.0475

### Morphological Measurements Confirm That the Two Devices Are Equivalent

Selected traits of cows used in trials 1, 2, and 3 are presented in Table 2. All these average values were determined from 3D images recorded from Morpho3D device, as it was considered as the reference method in these trials. Surface areas and volumes were 7.20 m^2^ and 764 L in trial 1, 7.18 m^2^ and 736 L in trial 2, and 6.28 m² and 581 L in trial 3, respectively. The height at the withers in trials 1, 2, and 3 was 1.45, 1.50, and 1.38 m, respectively (Table 2). A notable difference (about 0.08 m) in height at the withers (WH) was observed between the two devices (Table 3). For the other measurements, the differences between Morpho3D and Deffilait3D were of lesser magnitude (0.013 m for HW, 0.004 m for WB, 0.025 m for CD, 0.007 m for AC), and represented less than a 4% difference. For TV, only 1.2 L of difference was detected between the two devices, which is negligible compared to the volume of a cow (about 750 L). For the total surface area, the values obtained by the two devices differed by 0.6 m², i.e., about 8%.

**Table 2.** Average values of selected traits measured on 3D images of animals recorded with used Morpho3D

Trial number	TS, m²	TV, L	WH, m	HW, m	WB. m	CD, m	AC, m
1 (*n* = 16)	7.202	763.9	1.454	0.575	0.537	0.811	2.632
2 (*n* = 15)	7.181	739.2	1.499	0.565	0.530	0.787	2.587
3 (*n* = 13)	6.278	581.2	1.376	0.507	0.507	0.755	2.324

TV, total volume; TS, total surface; WH, withers height; HW, hip width; WB, buttocks width; CD, chest depth; AC, abdominal circumference.

**Table 3.** Comparison of the Morpho3D and Deffilait3D systems based on measurements obtained from 15 adult cows

	WH, m	HW, m	WB, m	CD, m	AC, m	TV, L	TS, m²
Morpho3D	1.540	0.578	0.526	0.813	2.580	739.8	7.481
Deffilait3D	1.459	0.565	0.530	0.787	2.587	738.6	6.881
Difference (in, %)	0.0811 (0.0053)	0.013.2 (0.0023)	0.0037 (0.0070)	0.0252 (0.0031)	0.0067 (0.0026)	1.2 (0.0016)	0.6 (0.0080)

WH, withers height; HW, hip width; WB, buttocks width; CD, chest depth; HG, heart girth; AC, abdominal circumference; TV, total volume; TS, total surface area.

**Figure 5.**
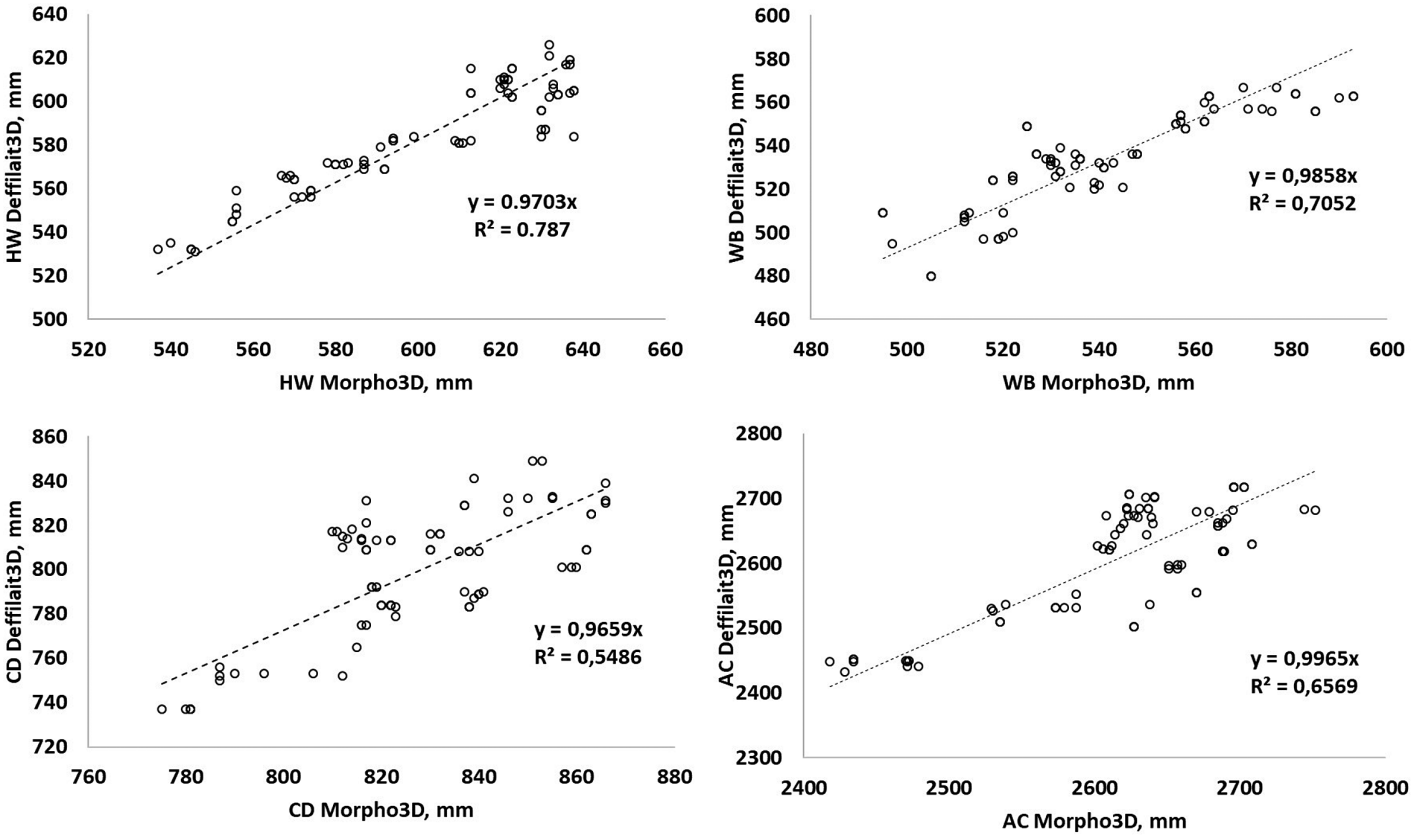
Comparison of measurements of hip width (HW), buttocks width (BW), chest depth (CD), and abdominal circumference (AC) between the Morpho3D and Deffilait3D devices (*n* = 200 measurements).

**Figure 6.**
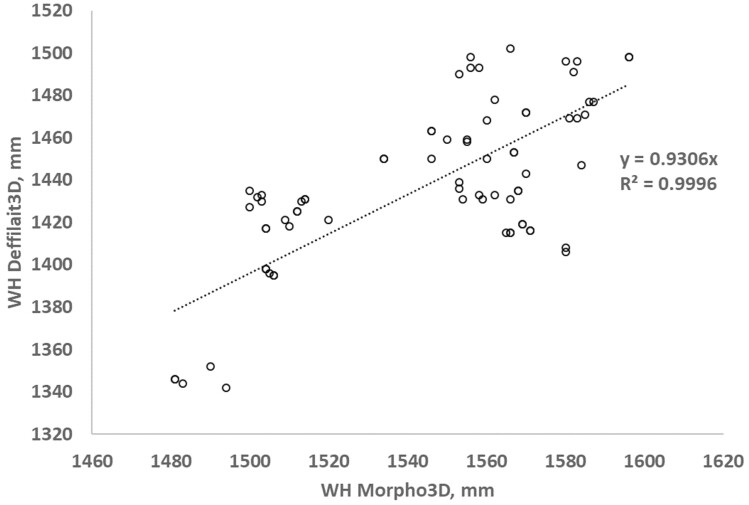
Comparison of measurements of withers height (WH) between Morpho3D and Deffilait3D (*n* = 200 measurements).

**Figure 7.**
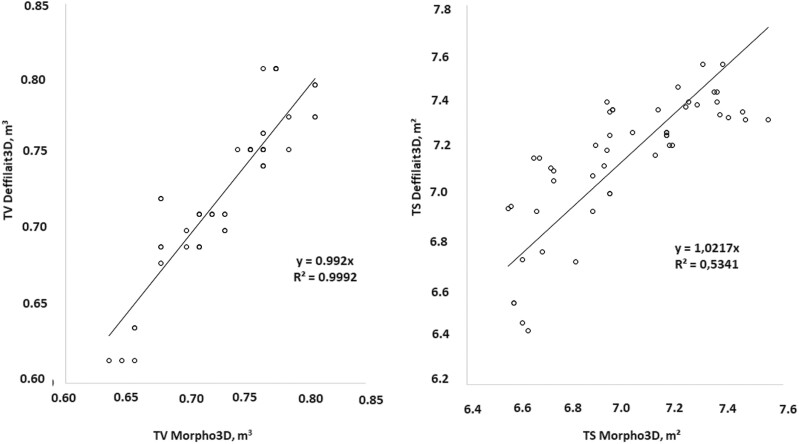
Comparison of measurements of total volume (TV) and total surface area (TS) between Morpho3D and Deffilait3D (*n* = 100 measurements).

To better illustrate the correlations between the measurements obtained using Morpho3D and Deffilait3D, a total of 200 measurements of HW, WB, CD, and AC were taken from images collected on the same cows with the two devices, and are compared in Figure 5. HW measurements were very similar between the two devices, with the R² and the coefficient of the equation (0.97) being close to one. For measurements of WB, CD, and AC, the *R*² values were lower (respectively, 0.70, 0.55, and 0.66), but with the coefficient of the equation again close to 1. In the case of WH, the *R*² coefficient was very good (0.99), but the coefficient of the equation was lower (Figure 6). This could be due to differences in the positions of animals during image acquisition (see discussion section). For TS and TV, for which comparisons were based on PS and PV, the values were largely similar between the two devices, but with some differences in TS. This was probably due to the smoothing procedure used for images from the Deffilait3D device, which requires further optimization (Figure 7).

### Application of the Device to Determine Animal Weight

**Figure 8.**
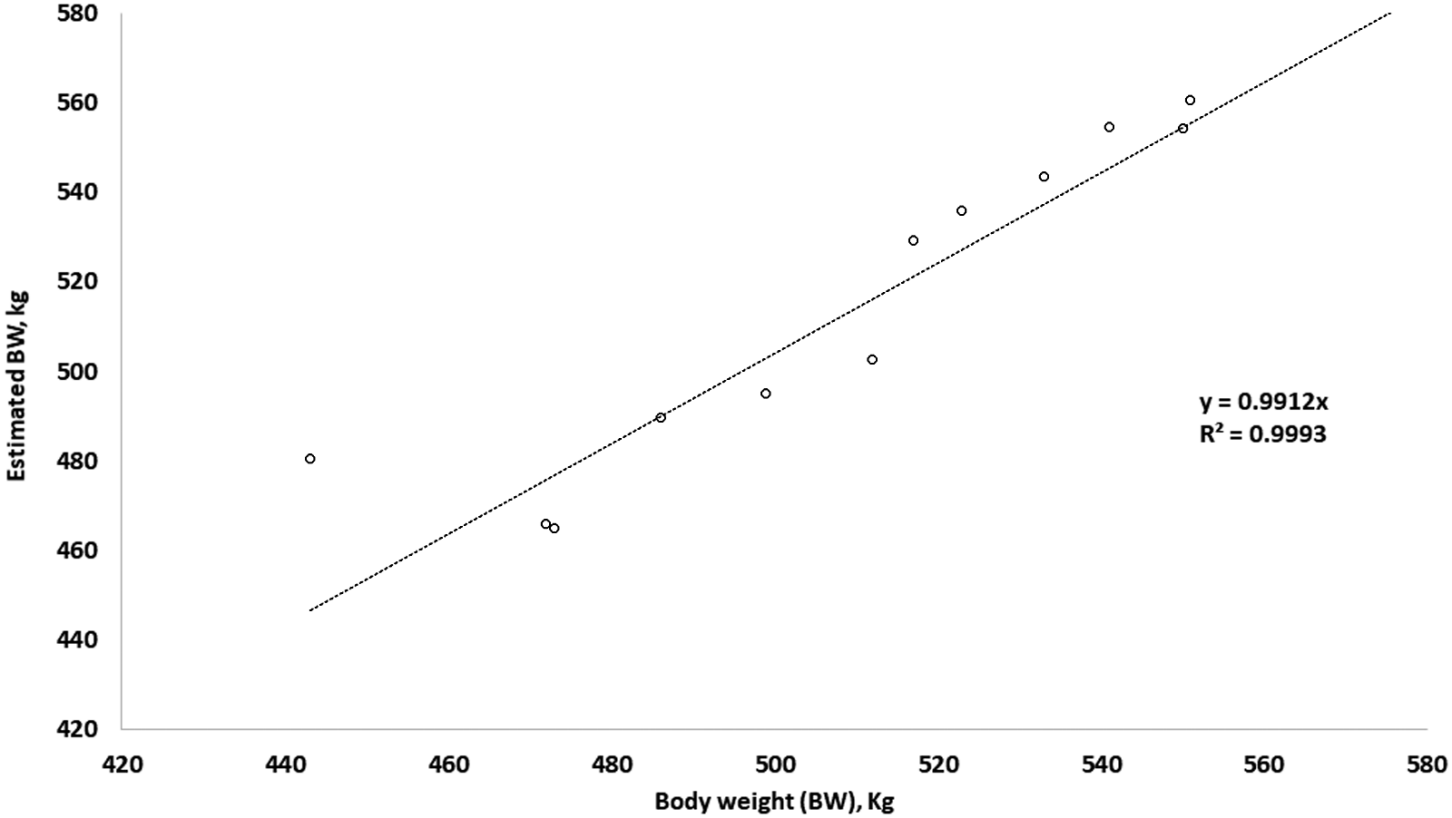
Comparison of body weight (BW) measured using a weighing scale with values estimated from the equation of Le Cozler et al. (2019a) using data from the Deffilait3D device. Estimated BW = 644 × TV + 408 × HW + 271 × WB—199, with BW, body weight; TV, total volume; HW, hip width; WB, buttocks width.

When we compared the measured values of BW to those that were predicted by the equation of Le Cozler et al. (2019a) based on data from the Deffilait3D device, we found that the *R*² coefficient was excellent (0.99), as was the coefficient of the equation (Figure 8).

## Discussion

The device, Deffilait3D, can be used to accurately measure or estimate, in a single shot, most morphological traits commonly used on farms. These data can then be combined for use in predicting body 7.

Deffilait3D was designed and developed for the high-throughput acquisition of both partial and whole 3D images, which allows work to be carried out on the entire animal or only a part of it (e.g., body condition score, at the back of the animal). Because our earlier device, Morpho3D, had been extensively validated in previous work (Le Cozler et al., 2019a), we used the measurements obtained from it as the reference values (“gold standard”) for the assessment of the Deffilait3D system. It should be noted, however, that the notion of a “gold standard” or “reference measurements” can be open to discussion: the fact that a value is used as a reference does not necessarily mean that it is more accurate or authoritative in all cases. Newly developed approaches and methods can sometimes give values closer to the true value than the reference method. This is especially the case for measurements for which the true value is notoriously difficult to determine, such as volume or surface area. Since 3D-imaging technologies have become available only recently, the methods used for morphological measurements have traditionally relied on the use of a tape (HG) and/or height gauge (WH, CD, etc.). In this trial, no manual measurements were performed because the Morpho3D measurement tool has been tested and validated for several years, and the resulting data are now considered as references.

For the majority of indicators, morphological measurements generated using the Deffilait3D device were repeatable and reproducible. For linear measurements (e.g., WH, HW), partial volume, and partial surface area, the repeatability coefficients obtained here ranged from 0.4% to 1.8% and were similar to those observed for the Morpho3D device (Le Cozler et al., 2019a). Even the highest CVs for repeatability and reproducibility, which were associated with complete volumes and partial areas, were still below the theoretical threshold of 4%, and should thus still be considered very good (Fischer et al., 2015). The slightly lower performance of the Deffilait3D device compared to its predecessor can probably be explained, at least in part, by the lower resolution of the one-shot acquisition strategy, which resulted in lower-quality 3D image reconstructions. This resulted in more artifacts (defects, small blisters, etc.) on images. However, during the experiment, we were able to make some improvements that resulted in improved image quality. Further calibration and modifications will make it possible to obtain smoother and better-reconstructed 3D images and will undoubtedly reduce measurement variability in the future.

Numerous studies have been carried out to estimate or perform body measurements in cattle (Auriol et al., 1961; Heinrichs et al., 1992; Tozser and Bedo, 2000), but few measurements have been performed using a 3D imaging device. In general, the main morphological measurements (HW, WB, TV, and TS) were very similar between the two devices compared here, with occasional minor differences (e.g., +0.4 cm between Deffilait3D and Morpho3D for WB). Point identification errors (on 3D images, in software such as Metrux6α®) seemed equally likely regardless of the device used. Although the results obtained with the two systems were largely comparable, the Deffilait3D has one considerable advantage: it is able to record the overall shape of an animal in only a few tenths of a second, compared with several minutes for Morpho3D. This new device therefore seems poised to make a significant contribution to the goal of high-throughput and accurate phenotyping. In the future, it may be possible to use successive images of the same animal, separated by a few tenths of a second, to generate dynamic data similar to what is possible with a strobe effect, but this remains to be further investigated.

The largest difference was found for measurements of WH, for which the two devices differed by about 8 cm. There are several possible explanations, including a distorted reconstruction of the underside of the legs with Morpho3D or a difference in the positioning of animals between the two devices. To address this, a “standard position” probably needs to be determined in the future, with which it may be possible to reduce the variation between devices. For example, Ling et al. (2022) highlighted the importance of a standard position in correctly determining body size measurements in pigs and Li et al. (2023) proposed a posture-based measurement adjustment method to improve accuracy measurements based on point-cloud data in beef cattle. In the Morpho3D device, the heads of animals are restrained thanks to the use of a self-locking head gate and therefore always raised, while with the Deffilait3D device, the animal’s head may move freely, which can affect leg position and thus, WH. Here, the average WH measured with the two devices was 154.0 and 145.9 cm, respectively. In an ongoing project using Deffilait3D device (Tiercin et al., unpublished data), wither height was corrected according to head and leg positions for a group of cows and the difference disappeared. Considering animal posture while registering 3D images is then of importance, at least for wither height measurement.

Using previously published equations validated in both adult and growing animals (Le Cozler et al., 2019b, 2022a), it was possible to determine the BW of cows. Here, we present the results of only one of the published equations, but results were similar to the others (not shown). In the future, the improved access to traits and/or indicators granted by the use of 3D imaging will likely make it possible to develop new equations that may be used even in the absence of such devices. In the meantime, the speed and ease of use of devices such as Deffilait3D may enable continuous monitoring of BW of a large number of animals throughout growth and/or adult life. As a step toward this goal, we are developing automatic extraction techniques based on a robust geometric algorithm as well as machine learning to improve automation of certain steps that must still be performed manually (e.g., preparing and “cleaning” the figures, determination of some measurements). Considerable progress has been made in the use of computer vision and deep learning methods, which are increasingly widespread in the field of agricultural data analysis (Kamilaris and Prenafeta-Boldu, 2018; Espejo-Garcia et al., 2019) and livestock production (Bezen et al., 2020). In the particular case of using convolutional neural networks, a dataset that includes both the image and its associated target variable is used to train the neural network. If the training is successful, the network can be used to create measurements from new data. convolutional neural networks can be used on depth maps but are unsuited for unordered point clouds (although networks for point clouds are emerging). Another approach is to provide the learning algorithm with a normalized representation of the 3D data, as described by Fischer et al. (2015).

Finally, for on-farm use, the number of cameras will decrease, as has been done for the Pheno3D device, which is a mobile device being tested since the beginning of 2023, on around 2000 growing beefs, from16 farms (Lebreton et al., 2023). Pheno3D is based on Deffilait3D development, using only 10 cameras, and costs around 18,000 US $. Preliminary results confirmed that this movable3D scanner allows high-throughput phenotyping of the whole cattle body for animals in motion, but also development of models to predict BW and the Linear score used by the French stakeholders to do selection on morphological criteria (Lebreton et al., 2023).

## Conclusion

Based on our results, the Deffilait3D device appears to be as accurate and precise as its predecessor, Morpho3D, in generating morphological data from cows, but can acquire images considerably faster. Measurements obtained with Deffilait3D were highly repeatable and reproducible for all of the studied traits, with coefficients of variation of less than 1.8% for repeatability and less than 3.3% for reproducibility. In the future, the development of automated reconstruction and analysis tools based on machine learning methods will facilitate improved extraction and/or analysis of indicators of interest at a very high rate, which will greatly increase the utility of this technology and its potential for widespread adoption in livestock farming or even on large wild animals. Such an approach opens the door to high-throughput acquisition of traits based on the whole animal as well as other partial measurements or analyses, such as rumen development, animal mobility, lameness detection, or body condition score.

## Supplementary Material

txae018_suppl_Supplementary_Files_1
