# Supplementary material for: One-shot technology for three-dimensional imaging of large animals: perspectives for ruminant management
Source: Transl Anim Sci. 2024 Feb 9;8:txae018. doi: 10.1093/tas/txae018 (PMC10896621; doi:10.1093/tas/txae018)
Supplement: txae018_suppl_Supplementary_Files_1 [file txae018_suppl_supplementary_files_1.docx]

Supplementary file 1: Repeatability (a) and reproducibility (b) tables used to validate the Deffilait3D device.

| a) | Total volume | Partial surface | Hip width | Wither height | Chest depth | Heart girth | Buttocks width | Diagonal length | Abdominal circumference |
| --- | --- | --- | --- | --- | --- | --- | --- | --- | --- |
| Error | 8.71 L | 0.13 m² | 0.0028 m | 0.0186 m | 0.0064 m | 0.0118 m | 0.0070 m | 0.0130 m | 0.0118 m |
| CV (%)* | 1.18 | 1.77 | 0.48 | 1.29 | 0.81 | 0.46 | 1.34 | 0.76 | 0.46 |
| CV 5328 (%)* | 1.44 | 1.37 | 0.10 | 0.32 | 1.23 | 1.06 | 2.93 | 0.97 | 1.06 |
| CV 5333 (%) | 0.00 | 0.36 | 0.60 | 0.32 | 0.69 | 0.18 | 0.77 | 0.24 | 0.18 |
| CV 6208 (%) | 0.00 | 0.50 | 0.09 | 0.89 | 0.54 | 0.23 | 0.41 | 0.43 | 0.23 |
| CV 6220 (%) | 0.76 | 2.32 | 0.99 | 3.07 | 0.05 | 0.04 | 1.70 | 1.74 | 0.04 |
| CV 7212 (%) | 0.83 | 0.57 | 0.31 | 1.62 | 0.47 | 0.32 | 0.26 | 0.38 | 0.32 |
| CV 7229 (%) | 0.00 | 0.90 | 0.18 | 0.22 | 0.00 | 0.37 | 0.89 | 0.63 | 0.37 |
| CV 7269 (%) | 0.69 | 1.56 | 0.32 | 0.14 | 0.79 | 0.48 | 1.27 | 0.15 | 0.48 |
| CV 8328 (%) | 2.86 | 4.05 | 0.28 | 0.28 | 1.48 | 0.19 | 0.33 | 0.15 | 0.19 |

| b) | Total volume | Partial surface | Hip width, | Withers height | Chest depth | Heart girth | Buttocks width | Diagonal length | Abdominal circumference |
| --- | --- | --- | --- | --- | --- | --- | --- | --- | --- |
| Error | , | 0.23 m² | 0.0064 m | 0.0264 m | 0.0116 m | 0.0470 m | 0.0100 m | 0.0447 m | 0.0475 m |
| CV (%) | 2.71 | 3.26 | 1.12 | 1.78 | 1.42 | 2.16 | 1.83 | 2.55 | 1.80 |
| CV 5288 (%)* | 3.45 | 4.23 | 0.83 | 1.04 | 0.80 | 2.07 | 2.04 | 1.58 | 1.87 |
| CV 6231 (%) | 3.70 | 3.74 | 0.55 | 1.80 | 1.97 | 3.73 | 1.17 | 2.56 | 2.46 |
| CV 6240 (%) | 2.60 | 2.58 | 1.71 | 1.24 | 1.42 | 1.58 | 2.68 | 1.33 | 0.54 |
| CV 7211 (%) | 2.80 | 3.31 | 1.33 | 2.85 | 2.26 | 1.77 | 2.27 | 2.77 | 1.81 |
| CV 7228 (%) | 2.81 | 3.36 | 1.00 | 1.51 | 0.24 | 0.87 | 0.89 | 0.81 | 1.50 |
| CV 7229 (%) | 2.28 | 4.31 | 0.89 | 2.44 | 1.76 | 2.40 | 1.21 | 4.40 | 2.23 |
| CV 7247 (%) | 1.44 | 4.08 | 1.29 | 1.34 | 0.51 | 2.22 | 1.23 | 2.74 | 1.72 |
| CV 7253 (%) | 1.52 | 2.54 | 0.90 | 1.25 | 1.20 | 1.20 | 2.28 | 2.71 | 1.75 |

CV: Coefficients of variation; * : number corresponds to cow
